# Assembly and Function of the Juxtaparanodal Kv1 Complex in Health and Disease

**DOI:** 10.3390/life11010008

**Published:** 2020-12-24

**Authors:** Delphine Pinatel, Catherine Faivre-Sarrailh

**Affiliations:** Institut de Neurobiologie de la Méditerranée, INSERM UMR1249, Aix Marseille Université, F-13273 Marseille, France; delphine.pinatel@inserm.fr

**Keywords:** myelin, node of ranvier, Juxtaparanode, Caspr2, Kv1 channels, axonal transport, axon initial segment, hyperexcitability

## Abstract

The precise axonal distribution of specific potassium channels is known to secure the shape and frequency of action potentials in myelinated fibers. The low-threshold voltage-gated Kv1 channels located at the axon initial segment have a significant influence on spike initiation and waveform. Their role remains partially understood at the juxtaparanodes where they are trapped under the compact myelin bordering the nodes of Ranvier in physiological conditions. However, the exposure of Kv1 channels in de- or dys-myelinating neuropathy results in alteration of saltatory conduction. Moreover, cell adhesion molecules associated with the Kv1 complex, including Caspr2, Contactin2, and LGI1, are target antigens in autoimmune diseases associated with hyperexcitability such as encephalitis, neuromyotonia, or neuropathic pain. The clustering of Kv1.1/Kv1.2 channels at the axon initial segment and juxtaparanodes is based on interactions with cell adhesion molecules and cytoskeletal linkers. This review will focus on the trafficking and assembly of the axonal Kv1 complex in the peripheral and central nervous system (PNS and CNS), during development, and in health and disease.

## 1. Introduction

The precise distribution of K^+^ channels at the axon initial segment (AIS) and the nodes of Ranvier is critical to ensure the appropriate initiation and faithful propagation of action potentials (APs) along myelinated axons. The AIS and nodes of Ranvier are fascinating structures to examine the mechanisms involved in neuronal polarity and subcellular patterning of axonal functional domains [1,2]. Membrane subdomains are distinctly segregated at the nodes of Ranvier with the node itself highly concentrated in voltage-gated Na^+^ (Nav) channels and the juxtaparanodes enriched in voltage-gated K^+^ (Kv) channels. These two domains are separated by the paranodal junctions anchoring the myelin terminal loops on both sides of the nodal gap. The mechanisms underlying the segregation of Nav1 channels at the AIS and nodes of Ranvier have been thoroughly examined in the PNS and CNS [3,4,5,6,7,8]. However, it is still unclear how the Kv1 channels are anchored at the AIS and juxtaparanodes. This review will focus on the dynamic processes that are involved in the subcellular targeting of the Kv1.1/Kv1.2 channels associated in complex with cell adhesion molecules and cytoskeleton linkers, including axonal transport, membrane diffusion and trapping, or internalization. A comprehensive view of the functional role of the diverse axonal K^+^ channels begins to emerge, taking into consideration the neuronal cell-type specificity. In pathological conditions such as demyelinating diseases in the PNS or CNS, disturbance of the nodes of Ranvier is associated with alteration of paranodal junctions and exposure of juxtaparanodal Kv1 channels that may contribute to neurological disorders.

## 2. Diversity of K^+^ Channels at the Axon Initial Segment and Nodes of Ranvier

The initiation and propagation of APs in myelinated axons depends on the high concentration of voltage-gated Na^+^ channels at the AIS and the nodes of Ranvier, respectively. A variety of K^+^ channels have been identified with precise axonal distribution to modulate neuronal excitability and the shape and frequency of APs. A common feature for myelinated axons both in the CNS and PNS is the presence of voltage-gated K^+^ channels including Kv1.1/Kv1.2 channels localized at the juxtaparanodes under the myelin sheath [9] and Kv7.2/Kv7.3 (also named KCNQ2/3) that are present at the node itself [10,11]. Kv1 and Kv7 family members are also found at the AIS regulating excitability at the site where APs are generated [12,13]. Moreover, other subtypes of axonal K^+^ channels are found along myelinated axons, with the voltage-gated Kv3.1b segregated at the nodal gap in a subset of CNS large myelinated axons [14]. The nodal K_Ca_3.1 channels activated by an activity-dependent influx of Ca^2+^, have been shown to secure continuous spike propagation in Purkinje cells [15]. The BK/K_Ca_1.1 channels, also activated by changes in membrane potential and intracellular Ca^2+^ concentration, localize to the paranodes solely in axons of cerebellar Purkinje cells to support the high-fidelity of AP firing at high frequency [16]. Recently, the two-pore-domain K^+^ channels TREK-1 and TRAAK, displaying thermal and mechanical sensitivity, have been identified at the nodes of Ranvier both in the CNS and PNS, and are not found at the AIS [17,18]. The K2P channels generate high-leak K^+^ current at the node, hyperpolarizing the membrane resting potential, thereby increasing Na^+^ channel availability for AP propagation [17]. As shown using pressure-patch-clamp recording at the nodes of Ranvier of trigeminal nerves, TREK-1 and TRAAK, most likely forming heteromers, drive rapid AP repolarization at the nodes and permit high speed and high-frequency AP conduction along myelinated axons [18]. In contrast, AP repolarization is not modified by TEA, a blocker of Kv1 and Kv7 channels, at the nodes of Ranvier of sensory nerves. Thus, AP repolarization may not mainly depend on voltage-gated K^+^ channels at the nodes of Ranvier as it occurs at the neuronal cell bodies.

The role of nodal Kv7.2/7.3 channels mediating a slow K^+^ current could be to finely modulate the excitability at the nodal region [19,20]. Moreover, voltage-gated K^+^ channels may finely tune excitability and secure axonal conduction at transition zones, including at branch points or in the region distal to the last myelinated segment near the nerve terminal [21,22]. The safety factor is altered at these sites because of impedance mismatch. Strikingly, recent reports indicate that myelination can be discontinuous in the cortical pyramidal neurons mostly in the superficial layers [23]. Non-uniform myelination is also found in parvalbumin GABAergic neurons in the cortex and hippocampus, which show irregular myelinated segments along with their branched axonal trees [24,25]. Transition zone excitability may have profound implications for signal integration in axonal trees. The precise distribution and function of the diverse K^+^ channels at these transition zones deserve further investigation.

The role of voltage-gated Kv1.1/Kv1.2 channels has been finely analyzed at the AIS where they are involved in the control of neuronal excitability, spike shape, and frequency. The low-threshold fast-activated Kv1 channels play a highly localized role in shaping the axonal AP at the AIS of cortical pyramidal neurons [26] or in dampening near-threshold excitability in fast-spiking cortical GABAergic interneurons [27], likely depending on Kv1 subunit composition in the different neuronal cell types. The slow inactivation of Kv1.2 enriched at the AIS of spinal motoneurons promotes the nonlinear spiking associated with the rhythmic locomotor activity [28]. However, their role at the nodes of Ranvier is a matter of speculation. The fast Kv1.1/1.2 channels are sequestered along myelinated axons on both sides of the node of Ranvier at the juxtaparanodes under the myelin sheath and do not normally influence AP repolarization. Kv1 channels are separated from the nodal gap by septate-like paranodal junctions anchoring the terminal myelin loops to the axolemma. The transmembrane septate-like junctions and the short paranodal width (3–5 nm instead of 10–20 nm at internode) are thought to reduce the current flows between nodal and internodal extracellular spaces. An intriguing observation is that the juxtaparanodal Kv1 channels are forming rosette particles aligned with Connexin29 channels in apposed myelin membrane as observed in freeze-fracture replica immunogold labeling of mouse sciatic nerves [29]. This would support a leak K^+^ conductance directly from juxtaparanodal axoplasm into the myelin cytoplasm that would not be dependent on voltage-gating. In the CNS, the inward-rectifying Kir4.1 channels are expressed by oligodendrocytes on the inner tongue of the myelin sheath facing the internodal axonal membrane. These channels ensure that the K^+^ released during neuronal firing is buffered by oligodendrocytes [30,31].

**Figure 1:**
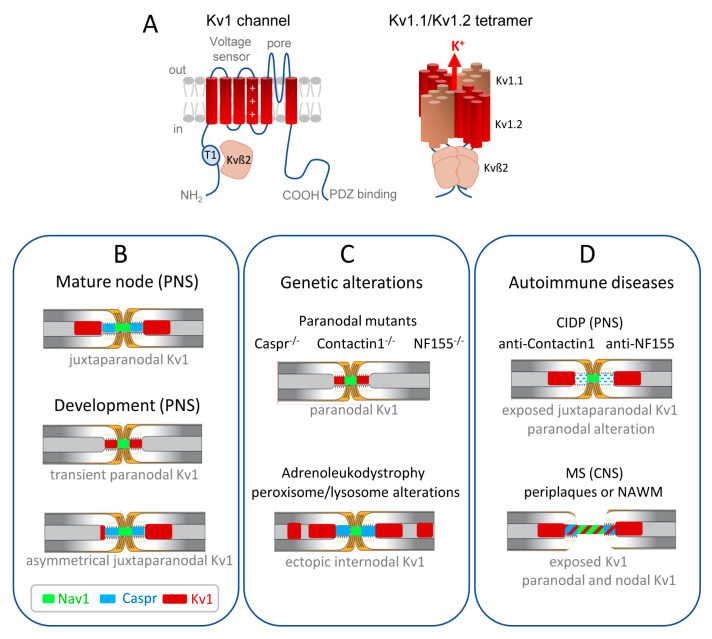
Distribution of the Kv1.1/1.2 channels in myelinated axons during development and misdistribution associated with genetic or autoimmune diseases. (**A**) (**Left**) Transmembrane topology of one Kv1 channel α-subunit, with the voltage-sensing module comprising transmembrane segments S1–S4 with the positive charges shown in S4, and the pore module comprising S5–S6. The T1 tetramerization domain is located in the N-terminal tail and the C-terminus contains a binding site for PDZ-proteins. The Kv1 α-subunit associates with an auxiliary Kvß2 subunit that is essential for regulation via its T1 linker. (**Right**) Juxtaparanodal Kv1 channels are composed of heterotetramers of Kv1.1 and Kv1.2 α-subunits forming the pore of the channel co-assembled with four Kvß2 subunits. (**B**) The Kv1 channels are transiently present at the paranodes in immature nerves when the axo-glial septate-like junctions are not fully established. In the PNS, the juxtaparanodes are first assembled asymmetrically before being settled on both sides of the paranodes. (**C**) Kv1 channels are mislocalized at the paranodes when the septate-like junctions are disrupted, as shown in mice deficient for the cell adhesion molecules, Caspr, Contactin, or Neurofascin155. Ectopic internodal clusters of Kv1 channels are observed associated with the alteration of myelin ganglioside in a mice model of X-adrenoleukodystrophy. (**D**) In autoimmune demyelinating neuropathy (CIDP), a subset of patients produce anti-Contactin or anti-Neurofascin155 IgG4 that induce the selective loss of paranodal transverse bands, but the distribution of Kv1 is unknown. In multiple sclerosis (MS) patients, early paranodal alterations occur as observed at the border of MS lesions in periplaques and normal-appearing white matter (NAWM), with the Kv1 channels abutting or even overlapping the nodal region.

Kv channels are the most diverse family of voltage-gated ion channels in vertebrates of which a significant proportion plays a critical role in controlling neuronal excitability and is of major relevance to neurological diseases. In particular, mutations of genes encoding Kv1 and Kv7 subfamily channels have been associated with a range of epilepsies and encephalopathies. Their pathogenic role is complex and partly defined by the axonal distribution of those channels [11,32,33]. This review will particularly focus on the role of Kv1.1/1.2 channels in physiological conditions and de- or dys-myelinating autoimmune diseases. The Shaker-related Kv1 subfamily comprises at least eight members (Kv1.1–Kv1.8). The α-subunits from the same subfamily assemble to form functional homo- or hetero-tetrameric channels (Figure 1A). The juxtaparanodes of myelinated axons contain generally heteromeric Kv1.1 and Kv1.2 channels associated with Kvß2 auxiliary subunits. The physiological role of these channels may be critical during development as their mature localization pattern is not achieved at the onset of myelination. Kv1.1/ Kv1.2 are first present at the node and paranodes in immature nerves when the axo-glial septate-like junctions are not fully established (Figure 1B) [34,35,36]. This transient localization may prevent aberrant excitations during the transition between continuous to saltatory conduction. Importantly, the juxtaparanodal Kv1 channels may also play a critical function in pathological situations when the paranodal junctions are altered and the myelin sheaths retracted (see Section 7).

## 3. Targeting of the Kv1 Complex at The Axon Initial Segment

**Figure 2:**
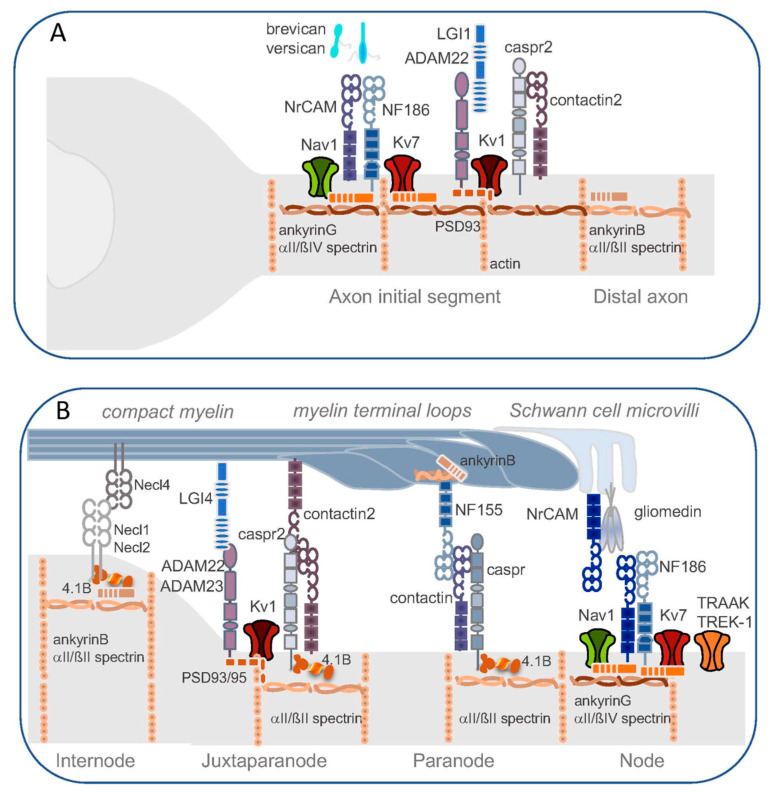
Molecular organization of the AIS and peripheral node of Ranvier. (**A**) Organization of cell adhesion molecules and ion channels at the AIS. AnkyrinG and ßIVspectrin assemble as a submembrane scaffold that plays a critical role in the recruitment of the voltage-gated Nav1.6 and Kv7.2/7.3 channels at the AIS, together with the cell adhesion molecules Neurofascin186 (NF186) and NrCAM. The Kv1.1/1.2 complex is enriched at the AIS exhibiting a distal distribution and is associated with PSD93. Caspr2 and contactin2 are also enriched at the AIS. ADAM22 is required for the recruitment of LGI1, but is dispensable for the concentration of Kv1 at the AIS. (**B**) Distinct complexes of cell adhesion molecules and channels are segregated at the different domains of myelinated axons, the node of Ranvier, paranode, juxtaparanode, and internode as shown here for the PNS. At the nodal gap, the voltage-gated Nav1.6 and Kv7.2/7.3 channels are recruited via their ankyrinG-binding sites together with Neurofascin186 and NrCAM. Neurofascin186 is clustered at the node first through its interaction with gliomedin and NrCAM on Schwann cell microvilli. The two-pore-domain K^+^ channels TREK1/TRAAK are new players at the node. The voltage-gated Kv1.1/1.2 channels are localized at the juxtaparanodes associated in complex with cell adhesion molecules. The cis complex of Caspr2 and Contactin2 interacts in trans with Contactin2 on the myelin. ADAM22, ADAM23, and LGI4 have been also localized at the juxtaparanodes. The submembrane cytoskeleton of the juxtaparanodes includes 4.1B, αII/ßII spectrin, PSD93, and PSD95. At the internode, the axonal cell adhesion molecules Necl1 and Necl2 interact with 4.1B and serve as partners for glial Necl4.

The mechanisms that control the precise axonal distribution of ion channels have been the subject of intense investigations (Figure 2A). A common ankyrinG-based mechanism retains the Nav1 and Kv7 channels at the nodes of Ranvier and AIS. AnkyrinG is acting as a masterpiece in the organization of the cytoskeletal scaffold at the AIS, which is a unique region creating axonal polarity [37,38]. AnkyrinG repeats offer multiple binding sites to link ion channels in its membrane-binding domain and ßIV-spectrin via its spectrin-binding domain. The Nav1 α-subunits contain an ankyrin-binding motif located in the cytoplasmic loop II-III [39,40] and the Kv7.2/7.3 subunits show a nearly identical ankyrinG-binding motif in their C-terminal region [13]. Moreover, the cell adhesion molecules Neurofascin and NrCAM that are organizing an extracellular scaffold at the AIS and nodes of Ranvier, contain in their cytoplasmic tail a conserved FIGQY motif for interaction with distinct ankyrinG repeats. In contrast, the molecular mechanisms by which Kv1 channels are enriched in the distal part of the AIS and at the juxtaparanodes are still poorly understood. For a comprehensive review of the diversity of K^+^ channels and their targeting within specialized neuronal compartments, see Trimmer (2015) [11].

The axonal distribution of Kv1 was first characterized at the juxtaparanodes of myelinated axons [9]. The juxtaparanodal clustering of Kv1 requires the axo-glial interaction mediated by the cell adhesion molecules Caspr2 and Contactin2/TAG-1 [41,42], and the cytoskeleton adaptor 4.1B (see next Section 4). Afterward, the Kv1.2 channels were reported at the AIS of human cortical pyramidal neurons co-localized with Caspr2 [12] and in rat retinal ganglion cells [43], being restricted at the distal region of the AIS. A similar complex of cell adhesion molecules including Caspr2 and Contactin2 is found at the juxtaparanodes and AIS as reported in mouse cultured hippocampal neurons [44] and motor neurons [45]. However, none of these cell adhesion molecules are required for the recruitment of Kv1 at the AIS [44,45,46,47]. The components associated with Kv1 can be precipitated using α-dendrotoxin-mediated cross-linking from rat brain membranes [48] and have been identified by proteomic analysis or using autoantibodies mostly against Caspr2 and LGI1 from patients with hyperexcitability diseases such as limbic encephalitis [49,50]. These experiments revealed that the Kv1 multiprotein complex contains Caspr2 and Contactin2 that are present at the juxtaparanodes, and it also includes LGI1. LGI1 is a secreted protein of the LGI (Leucine-rich glioma inactivated) family consisting of an LRR (leucine-rich repeat) and an epilepsy-associated domain. LGI1 is known to interact with ADAM22 and ADAM23 that are transmembrane receptors of the ADAM (a disintegrin and metalloproteinase) family. The ternary complex featuring LGI1 is associated with pre-synaptic Kv1 channels forming a trans-synaptic bridge at the glutamatergic synapse and modulating synaptic strength [51,52]. ADAM22 has been identified as a component of the juxtaparanodes and is also enriched together with LGI1 at the AIS of hippocampal neurons [46,53]. However, ADAM22 promotes the recruitment of LGI1 at the AIS but is not required for Kv1 concentration at this site [53]. Interestingly, LGI1 is expressed at the AIS of hippocampal CA3 neurons and regulates AP firing by setting the density of the axonal Kv1.1. LGI1 deficiency results in increased intrinsic excitability by down-regulating the expression of Kv1.1/Kv1.2 via a post-translational mechanism [54].

The cytoskeleton adaptor 4.1B is a critical component of the juxtaparanodes whereas it does not appear to be concentrated at the AIS. However, 4.1B has been shown to interact with NuMA1 being involved in the AIS assembly but not maintenance, through the inhibition of membrane protein endocytosis [55]. The membrane-associated guanylate kinase (MAGuK) protein PSD93 is enriched both at the AIS and juxtaparanodes. Its interaction with the C-terminal region of Kv1 promotes the formation of channel clusters at the surface of transfected COS7 cells [44]. However, PSD93 and PSD95 are dispensable for Kv1.2 recruitment at the AIS [45,46]. Caspr2 contains a C-terminal PDZ type II-binding site and does not directly associate with PSD-93 and PSD-95. Caspr2 is capable of binding CASK and MPP-family members of PDZ proteins [56,57,58]. Interestingly, the cytoplasmic tail of Caspr2 can induce the recruitment of MPP2 and CASK at the AIS of hippocampal neurons correlated with the level of endogenous Kv1.2 enrichment [59]. The 4.1B and PDZ-binding regions of Caspr2 are both required for immunoprecipitating MPP2 and its recruitment at the plasma membrane of HEK cells, whereas only the 4.1B binding site is required for precipitating Kv1 [56,59]. Thus, 4.1 and MPP proteins may be involved as a scaffold to recruit or stabilize the transmembrane protein Caspr2 at the AIS.

Therefore, a variety of cell adhesion and scaffolding molecules are encountered at the AIS that are associated with Kv1 channels although none of these partners are strictly required for their trapping at that site. These components may finely regulate the Kv1 concentration, positioning, or biophysical properties at the AIS as reported for LGI1 [54]. Moreover, the AIS has been reported to be a dynamic unit regulating the intrinsic excitability of neurons during homeostatic plasticity [60,61] or in pathological conditions [62,63,64]. The AIS distribution of the Kv1 complex may be regulated depending on the neuronal cell type, differentiation stage, or activity to fine-tune neuronal excitability.

## 4. Cell Adhesion Molecules Mediate Kv1 Trapping at the Juxtaparanodes

The juxtaparanodes are membrane axonal domains that are enriched in Kv1.1/Kv1.2 channels located under the compact myelin at the borders of the paranodes (Figure 2B). In contrast with the paranodes that show a very characteristic ultrastructural feature with intermembrane transverse bands forming the septate-like junctions between the axolemma and terminal myelin loops, the juxtaparanodes do not display any noticeable junctional specialization. The mechanisms that promote the subcellular localization of Kv1 channels along myelinated axons are not completely understood. The first molecular components that have been identified at juxtaparanodes are a couple of axonal cell adhesion molecules, Caspr2 and Contactin2/TAG-1 [41,42], closely related with the paranodal components Caspr and Contactin. Contactins are GPI-anchored proteins containing Ig-like and Fibronectin type III domains and Caspr and Caspr2 are type-I transmembrane molecules belonging to the NeurexinIV family. Caspr2 and Contactin2 are interdependent for their accumulation at juxtaparanodes [1]. Contactin2 is both expressed on the axonal and myelin sides and may be sufficient on the glial side only to foster the juxtaparanodal clustering of Kv1 channels [65]. The phenotype of the knock-out mice for Contactin2 or Caspr2 is not associated with the complete loss of Kv1 at juxtaparanodes but rather with a strong decrease in Kv1 channel concentration at the juxtaparanodes both in the PNS and CNS. More precisely, in the Contactin2 KO mice, Kv1.2 is missing in 70% of the juxtaparanodes of the sciatic nerves and the Kv1.2 immunoreactivity occupies much-reduced areas abutting the paranodes in the optic nerves [41]. The phenotype seems to be quite similar in Caspr2-deficient mice with the loss of Kv1 immunoreactivity in most of the juxtaparanodes of the sciatic and optic nerves as reported by Poliak et al. (2003) [42] or a severe reduction of Kv1 concentration in most of the juxtaparanodes in the spinal cord and sciatic nerve depending on the age examined between P20 and P180 [66]. In myelinated peripheral axons, the Kv1 channels associated with Caspr2 and Contactin2 are also found along the two lines flanking the mesaxonal line affixed to the inner lip of the myelin sheath along the internodes and below the Schmidt–Lanterman incisures where they may attenuate current leakage [67]. The Kv1 channels are preserved along the mesaxon in the Caspr2 KO mice and are only altered in the double Caspr/Caspr2 KO mice forming aggregates along the internodal axolemma in the sciatic nerve [68]. Since Caspr and Caspr2 share the ability to interact with similar adaptors, band 4.1B and ßII-spectrin, to link the cortical actin cytoskeleton, we can hypothesize that they may play a redundant function in the organization of the mesaxonal line. However, the accumulation of Kv1.2 at juxtaparanodes is severely altered in the 4.1B-null mice whereas Kv1.2 is properly localized along the mesaxon with a possible compensation by the 4.1R homolog [68]. Another adaptor of the protein 4.1 family, 4.1G, is implicated on the glial side in the organization of the internodal membrane by targeting and/or stabilizing the glial cell adhesion molecule Necl4/ SynCAM4 along the axo-glial interface. In the peripheral nerves of 4.1G KO mice, the Kv1.2 channels, Caspr2, and Contactin2 are not found as a double-strand along the mesaxon nor at the Schmidt–Lanterman incisures as observed in the wild-type axons and are aberrantly concentrated at the juxtaparanodes and adjacent areas. The presence of Caspr, Contactin, and NF155 is also affected along the mesaxon and not at the paranodes [69].

The clustering of Kv1 channels depends on neuron-glia interactions at juxtaparanodes mediated by Caspr2 and Contactin2. However, the presence of residual Kv1 expression in the Caspr2 or the Contactin2 KO mice may indicate some redundant mechanisms or neuronal cell-type specificity for the Kv1 clustering at juxtaparanodes. Another complex of cell adhesion molecules associated with the Kv1 channels may play a role in the assembly of juxtaparanodes, namely the ADAM22 and ADAM23 transmembrane receptors known to interact with secreted proteins of the LGI family. Interestingly, co-immunoprecipitation experiments showed that Caspr2 and Contactin2 associate with ADAM22 and ADAM23 indicating cross-interactions between these distinct Kv1-associated partners [53]. LGI1 and ADAM22 are enriched at the AIS modulating Kv1 channel expression to tune intrinsic excitability [53,54]. ADAM22 is enriched at the juxtaparanodes recruiting the MAGuK PSD-93 and PSD-95, but it is not required for Kv1 or Caspr2 accumulation within this subdomain [46,70]. On the other hand, axonal ADAM22 binds LGI4 expressed by Schwann cells to participate in axon-glial cell communication and is crucial for axonal sorting and myelination during PNS development (but not CNS) [71,72]. ADAM22-null mice have profound hypomyelination in the PNS [73]. The ADAM23 protein that is expressed in neurons and Schwann cells is not required for myelination but as observed for ADAM22, it accumulates in the juxtaparanodal domain in the PNS [74]. ADAM23 KO mice are characterized by a premature death during the second postnatal week and conditional mutants have been generated that will allow examining its role at the juxtaparanodes [74]. Our preliminary data indicate that conditional deletion of ADAM23 in parvalbumin-positive neurons strongly alters the Kv1.2 juxtaparanodal recruitment in hippocampal inhibitory neurons as also observed in the PNS (D. Meijer and C. Faivre-Sarrailh, unpublished observations).

The polarized distribution of the juxtaparanodal components at the internodal endings is critically dependent on the integrity of the paranodes. In myelinated axons with disruption of axo-glial septate-like junctions at paranodes, as occurring in mice deficient for either Caspr, Contactin, or NF155, the Kv1 channels relocate from the juxtaparanode to the paranodal region flanking the nodal gap [75,76,77,78]. This lateral displacement is observed for the entire Kv1 complex including Caspr2, Contactin2, and ADAM22 [46]. Therefore, the paranodal axo-glial junctions may act as a fence to exclude the juxtaparanodal membrane components, keeping the Kv1 channels separated from the nodal Nav1 channels (Figure 1C).

## 5. Organization of the Submembrane Cytoskeleton at Paranodes and Juxtaparanodes

Besides the role of axo-glial interactions, the cortical cytoskeleton plays an important role in the segregation of membrane proteins at the different subdomains of the node of Ranvier (Figure 2B). Band 4.1B belongs to a family of proteins that anchor membrane receptors to the cortical actin/spectrin cytoskeleton. 4.1B is localized all along myelinated axons except at the nodal gap and is enriched at paranodes and juxtaparanodes through Caspr and Caspr2 binding, respectively [79,80]. 4.1B also interacts with nectin-like proteins (Necl1 and Necl2 also known as SynCAM3 and SynCAM1) along the internode [81,82]. Three different lines of 4.1B-null mice have been generated independently and the most striking phenotype was the frequent loss of the juxtaparanodal domain in both the CNS and PNS with an absence of Kv1.2, and a marked reduction of Caspr2 and Contactin2 immunostaining [83,84,85,86,87]. Destabilization of the paranodal junctions was also reported that may be associated with the loss of transverse bands [85,86]. A differential expression of 4.1B is reported in the CNS and PNS with the 145/125 KDa splice variants expressed in the spinal cord and brain, and multiple isoforms in the sciatic nerve [85,86]. This may account for the discrepancy between the phenotypes reported for the different mutant lines, with some residual expression of truncated 4.1B isoform in some of the knock-out mice [84,85]. The concentration of cortical αII- and ßII-spectrin appears markedly reduced along the internode in the sciatic nerves of 4.1B-deficient mice and mistargeting at the nodal gap was also observed [84,85]. This is a strong indication that 4.1B mediates anchoring with the spectrin cortical cytoskeleton in myelinated axons as an important mechanism for the compartmentalization of cell adhesion molecules and associated ion channels along the axonal membrane. The phenotype of mice expressing Caspr or Caspr2 deleted from its 4.1B biding site was also examined in the corresponding null background [83]. As expected, Caspr2 has a 4.1B binding site that is necessary for its accumulation at juxtaparanodes. In contrast, Caspr deleted from its 4.1B binding site is still recruited at the paranodes indicating that the assembly of axo-glial contacts at paranodes is mostly based on extracellular interactions. However, the paranodal fence function is altered with Kv1 not efficiently excluded from the paranodes.

The submembranous cytoskeleton (actin, ankyrin, spectrin) is organized with a periodic spatial arrangement along the unmyelinated axons. AnkyrinB and αII/ßII-spectrin define a boundary that may restrict ankyrinG and αII/ßIV-spectrin at the proximal region of the axon, the AIS [1]. The spectrin cytoskeleton also acts as a boundary at the nodes of Ranvier. As analyzed using stimulated emission depletion (STED) microscopy, the periodic organization of the cortical actin cytoskeleton is maintained at the transition between nodal ßIV-spectrin and paranodal ßII-spectrin. The Kv1.2 channels do not display a periodic organization at the juxtaparanodes even if the ßII-spectrin scaffold is still periodically organized as observed at paranodes [88]. The conditional ablation of ßII-spectrin in sensory neurons does not prevent the assembly of septate-like junctions at paranodes, but induces an alteration of the barrier that restricts the Kv1.2 channels at juxtaparanodes [89]. The Kv1.2 channels and associated Caspr2 and Contactin2 are found mislocalized at the paranodes and even at the node. Therefore, the paranodal submembrane scaffold acts as a border to prevent the lateral diffusion of the Kv1 membrane complex towards the node.

During development, Caspr2 and Kv1 are first enriched at the paranodes before they shift to distal paranodes and juxtaparanodes as observed in the CNS and PNS [35,36,90]. Therefore, the Kv1/Caspr2 complex should be initially tethered by 4.1B at paranodes together with the Caspr/4.1B complex. In mature myelinated axons, the lateral positioning of Kv1/Caspr2 complex to juxtaparanodes may be induced by their progressive exclusion from the rows of septate-like junctions when the paranodal loops become compact. An intriguing observation is that the assembly of juxtaparanodes is asymmetrical during PNS development (Figure 1B). The clustering of the Kv1 complex first occurs only at the distal juxtaparanode relative to the soma (or proximally relative to the internode) along the developing sciatic nerve [34]. Such asymmetry is observed as well in dissociated myelinating culture of dorsal root ganglia (DRG) indicating that this polarized distribution relies on an intrinsic mechanism rather than on a gradient controlling the proximo-distal polarity. This distribution may not depend on AP propagation since not inhibited by Tetrodotoxin (TTX)-treatment [36].

## 6. Trafficking and Axonal Transport of the Juxtaparanodal Components

Since the precise localization of axonal ion channels is crucial for their proper function, it is critical to understand how these ion channels, their auxiliary subunits, and associated cell adhesion molecules are transported and addressed along the myelinated axons [91,92]. Live-cell imaging of axonal transport of the node of Ranvier components is tricky in myelinated axons. Moreover, the assembly of juxtaparanodes is only established at mature stages in long-term myelinating cultures of PNS DRG [36,84] or CNS hippocampal neurons [47,93]. Therefore, the trafficking of the Kv1 channels and associated partners has been mostly examined in unmyelinated hippocampal neurons in culture focusing on clustering at the AIS and along the axon. Moreover, attention has been given to the comparison between the trafficking of nodal and juxtaparanodal components. A key question is whether the multiprotein channel complexes that are segregated at distinct membrane domains of myelinated axons are pre-assembled, transported, and inserted as a complex or whether they are transported independently and recruited only locally together. Pre-existing pools of cell adhesion molecules are diffusely present along the axon before myelination and become partitioned within distinct membrane domains by cognate glial ligands at the nodes, paranodes, or juxtaparanodes. Once myelination has been established, axo-glial septate-like junctions at the paranodes prevent the lateral diffusion of membrane cell adhesion molecules and channels [36,94]. Since then, the replenishment of all components during maintenance may require their sorting in axonal transport vesicles and docking into either the nodal or the internodal region, and/or clearance from the mistargeted domain. Classically, sorting of transmembrane cargoes into transport vesicles is thought to occur in the trans-Golgi network via signals in the cytosolic tail that are recognized by the various adaptor proteins and molecular motors.

A pioneering study from the lab of Salzer investigated the targeting of components to PNS nodes of Ranvier using viral infection in DRG explants and dissociated cultures [94]. Prior to myelination, the nodal cell adhesion molecule Neurofascin186 is diffusely expressed along axons and it is afterward trapped to the nodes by interaction with gliomedin expressed on Schwann cell microvilli. In contrast, the Nav1 and Kv7 channels associated with ankyrinG may require axonal selective transport before being recruited at the nodes. In mature nodes bordered by the paranodal septate-like junctions, Neurofascin186 and Nav1 channels are being delivered by vesicular transport and display a slow turnover for replenishment. The nodal Neurofascin186 and NrCAM are co-transported in the anterograde direction as reported in the PNS using DRG cultures at a stage of early myelination [95]. In contrast, these cell adhesion molecules seem to be independently transported from the beta subunits of the Nav1 channel (Navß1 and Navß2) or the Kv7.3 subunit, which are however similarly segregated at the nodal gap. The anterograde co-transport of Neurofascin186 and NrCAM depends on their interaction with ankyrinG. Interestingly, ankyrinG binds Kif5B/kinesin-1 and appears to be implicated in the transport of Nav1 channels into axons in cultured hippocampal neurons [96]. In another study, Neurofascin186 is observed to be partially co-transported with Navß2, with 50% of co-transport in the anterograde direction [97]. The transport of this pre-assembled complex is dependent on a major part of the molecular motors Kif5A and Kif5C [97]. As expected, the transport of Neurofascin186 occurs independently from the paranodal Caspr or internodal Necl1 which both interact with 4.1B and not with ankyrinG [95]. The paranodal components Caspr and Contactin, which are early associated in cis in the endoplasmic reticulum or their proper delivery at the cell surface [98], are co-transported in anterograde vesicles at a velocity ranging from 0.5-1.2 µm/s compatible with fast axonal transport as observed in cultured hippocampal neurons (our unpublished results).

Another intriguing question is how the cargo vesicles may recognize their site of detachment from microtubules prior to membrane insertion. The multi-binding site adaptor, ankyrinG, may be used for the Nav1 and Neurofascin186 vesicular transport along the microtubules by interacting with kinesins and also with the plus end microtubule tracking proteins EB1/EB3 [99]. AnkyrinG further mediates their trapping with the submembrane actin/spectrin scaffold at discrete regions of the axon including the AIS or more distally along myelinated axons, at the nodal regions. AnkyrinG repeats in the membrane-binding domain offer multiple binding sites and thus can link Nav1.2 with the Kif5 motor [96] and it interacts with spectrin via its spectrin-binding domain. After reaching the proper axonal site, ankyrinG has to dissociate from Kif5 to unload its cargo. The AIS enrichment of casein kinase CK2 might participate in such a process through the phosphorylation-mediated positive regulation of the interactions between Nav1 and ankyrinG [100]. CK2 is also present at the nodes of Ranvier in the PNS and CNS and may regulate the specific accumulation of Nav1. The anchoring of Nav1 and Neurofascin186 that relies on ankyrinG may prevent their endocytosis providing a powerful mechanism of retention within these domains [101].

**Figure 3:**
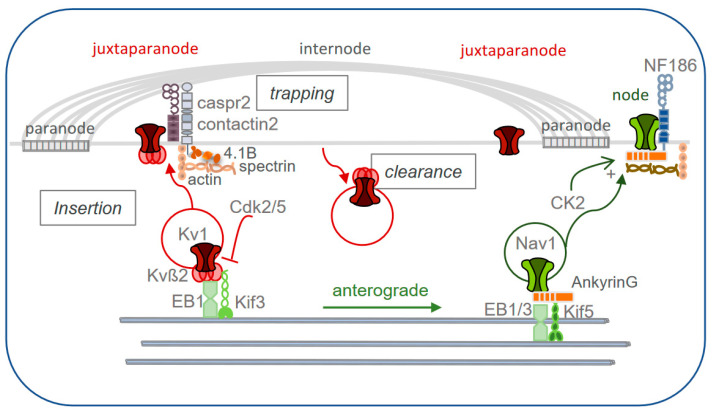
Trafficking and axonal transport of the juxtaparanodal components. The selective targeting of the nodal and juxtaparanodal ion channels requires their sorting in axonal transport vesicles and docking into either the nodal or internodal region, and/or clearance from the mistargeted domain. The paranoidal junctions are acting as a fence to prevent lateral diffusion. The Kvß2 subunits associated with the pore-forming a-subunits of Kv1 provide an interface with the microtubule motors Kif3 and EB1 for anterograde axonal transport. The Cdk-mediated phosphorylation of Kvß2 inhibits EB1 binding and may allow the docking at juxtaparanodes. The axo-glial cell adhesion molecules Caspr2 and Contactin2 further induce trapping and stabilization of the Kv1 complex. On the other hand, Nav1 associated with ankyrinG linking Kif5 is transported in a vesicle unloaded at the nodal gap. AnkyrinG may be used for recognition of detachment site and CK2-mediated phosphorylation may participate in such docking by reinforcing the interaction between Nav1 and ankyrinG.

The axonal targeting of the Kv1 channels at the AIS and juxtaparanodes is even more complicated due to the heteromeric assembly of Kv1.1, Kv1.2, or Kv1.4 α-subunits in tetramers forming the pore of the channels (Figure 1A). Moreover, the surface expression and axonal transport of the tetrameric Kv1.1/Kv1.2 channels depend on their association with the Kvß subunits. The Kv1.1 α-subunit is mostly ER retained and its heteromeric association with the Kv1.2 α-subunit promotes ER exit, cell surface expression, and specificity for axonal targeting. The Kv1.1/Kv1.2 subunits associate early in the ER via their T1 tetramerization domain with four Kvß subunits, mostly the Kvß2 isoform in the brain [102]. The Kvß2 subunits provide an interface to link with cargo adaptors and the microtubule motors thereby promoting axonal targeting of Kv1 channels (Figure 3). Kvß2 binds Kif3/kinesin-2 and EB1, both required for Kv1 channel axonal targeting [103]. An interaction of Kif5B with Kv1.2, likely by its T1 domain, has been also reported to be involved in the transportation and axonal targeting of the channel [104]. Kvß2 and EB1 closely associate with each other as they moved anterogradely along the axon as analyzed by Fluorescence Resonance Energy Transfer (FRET) and live-cell imaging. Interestingly, the interaction of Kvß2 with EB1 is negatively regulated by cyclin-dependent kinase (cdk)-mediated phosphorylation. The Kvß2 auxiliary subunit phosphorylated at serine 31 is co-localized with EB1 and cdk with enrichment at the AIS. Acute inhibition of cdk activity leads to the intracellular accumulation of EB1 and Kv1 channels at the AIS as an indication that cdk phosphorylation may signal to allow the detachment and docking of the cargo at the cell membrane when reaching its destination at the AIS [105]. Interestingly, cdk2 and cdk5 are also enriched at the juxtaparanodes in adult mouse sciatic nerve, as an indication that cdk-mediated phosphorylation may also regulate the targeting of Kv1 channels along myelinated axons.

The cell adhesion molecules associated with the Kv1 channels can be also transported as a pre-assembled complex. Pair-wise analyses indicate that the juxtaparanodal components Caspr2 and Contactin2 can be sorted within the same axonal transport vesicles in the anterograde direction in hippocampal neurons [59]. However, it should be noted that they are differentially distributed along the axon with Contactin2 being strongly enriched at the AIS and Caspr2 more uniformly distributed along the axon as an indication that different mechanisms may occur after their insertion at the axolemma including diffusion/trapping or internalization events. In the same manner, ADAM22 and ADAM23, which localize at the juxtaparanodes and interact with Contactin2 and Caspr2, are co-transported anterogradely together with Contactin2 or Caspr2 [53]. However, it is not known whether the juxtaparanodal cell adhesion molecules are transported by Kif3/kinesin-2 as reported for the Kv1 channels. Moreover, we do not know whether the adaptor 4.1B, which is critically implicated in the segregation of the Kv1 multimolecular complex at juxtaparanodes is also co-transported with the membrane components and plays a role as an adaptor during vesicular transport.

Another important mechanism that favors selective axonal targeting is the clearance from the mistargeted domain. As a matter of the fact and in contrast to the Kv1 channels, Caspr2 does not exhibit polarized vesicular trafficking and is transported in the anterograde direction both in axon and dendrites in cultured hippocampal neurons. The selective endocytosis of Caspr2 is achieved in the somato-dendritic compartment based on the PKC-phosphorylation of a short motif in the 4.1B binding region [106]. Caspr2 can be also targeted to endosomes in dendrites via its C-terminal PDZ-binding domain interacting with PAR3 [107]. Since PAR3 has been shown to interact with the kinesin Kif3A [108], Caspr2 may be axonally transported through the Kif3 motor after its somato-dendritic internalization. The dynamic clustering of Kv1 channels has been analyzed using a myelinating co-culture of hippocampal neurons and oligodendrocytes [93]. The cell adhesion molecule Contactin2 is implicated in the internodal clustering of the Kv1 channels by mediating the phosphorylation of Kv1.2 at tyrosine458. The trans-homophilic interaction of Contactin2 may recruit Kv1 channels into the lipid rafts, favoring tyrosine phosphorylation and anchorage to the actin cytoskeleton, thereby preventing its internalization at discrete sites along the internode.

The Kv1.1/1.2 channels are associated with their ß2 auxiliary subunits early during the secretory pathway and are sorted in axonal transport vesicles via Kif3/kinesin-2 and EB1. The cell adhesion molecules that are components of the juxtaparanodal complex (Contactin2, Caspr2, ADAM22, and ADAM23) may associate in cis-complex moving in vesicles selected for anterograde axonal transport. After insertion along the axolemma, the contact with myelin ligands within the internode may induce their progressive trapping and stabilization at the juxtaparanodes and clearance from unappropriated membrane localization (Figure 3).

## 7. The Kv Channels in De- or Dys-Myelinating Neuropathy May Participate to Alterations of Axonal Conduction

The distribution of Kv1 channels is strongly modified in myelinated axons with defective paranodes, as observed in mice deficient for the cell adhesion molecules Contactin, Caspr, or Neurofascin155 (Figure 1C) [75,76,77,78]. The disruption of paranodal junctions allows the lateral diffusion of Kv1.1/Kv1.2 in the vicinity of the nodal gap so that they become overexposed leading to perturbation of conduction. Mice with a null mutation of the ceramide-galactosyl-transferase (CGT) or the cerebroside sulfotransferase (CST) that perturbs the myelin glycolipid environment show preserved compact myelin and abnormal paranodes, which is correlated with the movement of Kv1 channels towards the nodal gap [109,110]. Similarly, in the dys-myelinating jimpy mutant mice, the Kv1 channels are located at paranodes adjacent to nodal clusters at P15 and become diffuse at later stages [111]. Moreover, alterations of the axo-glial paranodal junctions allow the passage of the extracellular current flow towards the internode. Interestingly, in the double Caspr/Caspr2 mutant, Kv1 are absent from the paranodes and nevertheless, the nerve conduction velocity is not different from that of the single Caspr mutant [68], reflecting the large effect that the decrease in paranodal resistance has on conduction velocity [112]. Blocking the function of Kv1 channels when the paranodal junctions are disrupted, results in an increase in the amplitude and refractory period, but not in conduction velocity [76]. Therefore, the misdistribution of Kv1 channels when the paranodes are compromised could contribute to the loss of AP propagation in de- or dys-myelinating pathologies [113].

Whether the node of Ranvier is an initial target in demyelinating pathologies is an important issue. The pathogenic role of autoantibodies directed against nodal and paranodal components has recently emerged in the field of chronic inflammatory demyelinating polyradiculoneuropathy (CIDP) [114,115,116,117]. Peripheral nodo-paranodopathies in a subset of CIDP patients are associated with autoantibodies against Contactin or Neurofascin inducing the disruption of the paranodal transverse bands as observed in nerve biopsies (Figure 1D) [118,119]. To my knowledge, whether as a consequence, the Kv1 channels become mislocalized to paranodes is not documented at present. CNS demyelination and dysmyelination pathologies are associated with a major modification of nodal Nav channels [120]. In multiple sclerosis (MS) demyelinated lesions, disruption of the nodes is associated with heterogeneous distribution of Nav channels with a diffuse immunoreactivity and also few broad aggregates and diffuse distribution of paranodal and juxtaparanodal components. Loss of juxtaparanodal clusters is observed with extensive expression of Kv1.2 along denuded axons [121]. It seems that early paranodal alterations occur at the onset of myelin damage as observed at the border of MS lesions, with disrupted Neurofascin155 paranodal structures and movement of Kv1 channels abutting or even overlapping the nodal region, before the disruption of the node itself (Figure 1D) [122,123]. In the Shiverer mutant mice used as a non-injured model of dysmyelination, the absence of the structural myelin protein MBP prevents myelin compaction and assembly of paranodes [124]. The Kv1.1/1.2 channels display increased and diffuse expression reducing the amplitude of compound APs in the spinal cord axons as shown using 4-aminopyridine (4-AP) and dendrotoxin-I blockers [125]. Similarly, alterations of the juxtaparanodal domains have been reported in experimental autoimmune encephalomyelitis (EAE), a model of inflammatory CNS demyelination, and in the cuprizone model of toxic demyelination [126,127]. In the corpus callosum, inflammation alone is sufficient to induce the diffusion of Kv1 channels and the reorganization of juxtaparanodes is a later event during remyelination that may remain incomplete with the persistence of an inflammatory environment [126]. Hence, the Kv1 channels that become exposed at paranodes and internodes may contribute to an alteration of AP propagation in MS. Blockade of Kv1 channels with 4-AP is used as a symptomatic treatment in MS to broaden APs and improve neuromuscular transmission [128,129]. However, 4-AP may also directly target the pre-synaptic high voltage-activated Ca^2+^ channels to potentiate neurotransmitter release [130].

As another consequence of ion channel dispersion induced by demyelination, diffuse axonal expression of Kv7.3 subtype is observed in cuprizone-demyelinated cortical neurons that may induce periaxonal K^+^ accumulation and trigger ectopic APs. Such axonal hyperexcitability may induce alterations of the computational functions of myelinated pyramidal neurons [131]. This K^+^ channel may represent an alternative to exploring new pharmacological therapies for the cognitive symptoms associated with demyelinating CNS disorders. Moreover, the role of the recently identified nodal TRAAK/TREK1 channels in demyelinated axons remains to be investigated.

The role of Kv1 channels has been highlighted when mis-distributed in peripheral neuropathies. Neurometabolic genetic diseases with peroxisomal dysfunctions, such as X-linked adrenoleukodystrophy (X-ALD), can be associated with inflammatory cerebral demyelination and severe neurological deterioration or myelopathy and peripheral neuropathy. In a mouse model with Schwann cell deletion of the peroxisomal biogenesis factor peroxin 5 (PEX5), it was recently reported that disrupted peroxisomes induced the failure of lysosomes thereby impairing the turnover of gangliosides in peripheral myelin [132]. GM1, normally enriched at the paranodes, and GD1a are dispersed into the internodes in the mutant nerves. Nerve conduction velocity and compound APs are diminished in mutants without dysmyelination, axonal loss, or paranodal alteration in the sciatic nerve. Strikingly, the PEX5 mutant shows ectopic internodal clusters of Kv1.1/Kv1.2 channels associated with Caspr2 and Contactin2 (Figure 1C) [132]. A similar phenotype is also observed in the other models of peroxisomal dysfunctions (ABCD1- or Mfp2-deficient mice). The glycolipids, which are associated with the lipid rafts, may be implicated in the trafficking and topographical organization of membrane proteins at paranodal and also juxtaparanodal axo-glial contacts [133,134,135]. In the PEX5 mutant nerve, the ectopic distribution of Contactin2, which is a GPI-anchored glycoprotein associated with the lipid rafts, could be induced by the perturbed ganglioside environment, drifting into internodes in complex with Caspr2 and Kv1. The opening of ectopic internodal Kv1 channels may contribute to a disturbed equilibrium of axonal ions causing slowed nerve conduction velocity and conduction blocks.

The modulated expression of Kv1 channels has been also investigated in neuropathic pain following peripheral nerve injury suggesting possible ways of treatment. An ectopic activity develops in myelinated nerves after injury arising both at the neuroma site and at the level of DRG. Although the role of Kv1 channels is uncertain at the juxtaparanodes of the naïve peripheral nerve, they may protect the nerve from hyperexcitability after axotomy. The distribution and composition of Kv1 channels are modified after sciatic nerve axotomy in the rat with decreased Kv1.2 and expression of Kv1.4 and Kv1.6 at the juxtaparanodes. Moreover, alteration of paranodes with the loss of ßII-spectrin results in the misdistribution of Kv1 at the paranodes. The paranodal expression of Kv1.4 and Kv1.6 is also observed in tissue resected during surgery from patients with painful Morton neuroma [136]. The Kv1 channels may fulfill an adaptive role in suppressing excitability in myelinated afferents following traumatic nerve injury.

Peripheral nerve hyperexcitability associated with altered juxtaparanodal Kv1.2 was reported in patients suffering from type 2 Diabetes Mellitus [137]. In the diabetic db/db mice, a reduced activity of fast Kv1-current is involved in the hyperexcitability phenotype. In the sciatic nerve, a strong reduction of juxtaparanodal Kv1.2 channels is observed whereas Kv1.1 and Kvß2 subunits are not affected. The expression level of Kv1.2 is normal indicating that most probably its anchoring at juxtaparanodes may be altered, but without accumulation in the soma or any demyelinating phenotype. A reduction of juxtaparanodal Kv1.2 was also observed selectively in ventral roots from autopsy cases of sporadic amyotrophic lateral sclerosis (ALS) [138]. Extensive fasciculations are prominent features of ALS presumably mediated by increased motor neuron excitability that occurs as a precursor of motor neuron death. Besides excitotoxicity, axonal excitability has been associated with increased persistent Na^+^ currents and reduced fast K^+^ currents in ALS patients that may be correlated with reduced mRNA expression of Kv1.1, Kv1.2 and Kv7.2 and impaired axonal transport. These results raised the possibility of treatments with the K^+^ channel openers such as Retigabine that could decrease axonal excitability.

## 8. The Kv1 Complex Components Caspr2 and LGI1 Are Target Antigens in Autoimmune Diseases Associated with Hyperexcitability

The voltage-gated Kv1 channels (VGKC) are involved in three autoimmune diseases associated with hyperexcitability [139]. Limbic encephalitis is characterized by CNS symptoms, neuromyotonia by peripheral nerve hyperexcitability and Morvan’s syndrome is presenting a combination of neuromyotonia with autonomic and CNS involvement. Actually, Caspr2 and LGI1 are the main target antigens implicated in these syndromes giving insights into the pathogenic mechanisms associated with alterations of the Kv1 complex. Limbic encephalitis may include cognitive impairment, memory loss, hallucinations, and seizures with distinctions between patients with anti-Caspr2 or anti-LGI1 autoantibodies [140,141,142]. As a shared feature, Caspr2 and LGI1 belong to the VGKC complex although the mechanisms associated with their pathogenicity leading to central hyperexcitability may differ. LGI1 is expressed in the CNS and present at the AIS and at the glutamatergic synapses. Patient-derived antibodies against LGI1 were found to inhibit interactions between LGI1 and ADAM22 or ADAM23 and to decrease synaptic AMPA receptors clustering altering hippocampal long-term potentiation [143,144]. Infusion with anti-LGI1 antibodies also causes decreased expression of axonal Kv1.1 associated with hyperexcitability [54,144]. Whether the passive transfer of anti-Caspr2 patient antibodies induces alteration of Caspr2 expression in mouse brain is uncertain and may depend on the route of injection [145,146,147]. Intracortical injection of anti-Caspr2 antibodies has been reported to decrease the dendritic levels of Caspr2 altering synaptic AMPA receptor trafficking and leading to perturbation of excitatory transmission in the mouse visual cortex [146]. Intraperitoneal injection of anti-Caspr2 antibodies in mice induces behavioral alterations with mild working-memory defects and limited social interactions. In this last study, the brain deposition of anti-Caspr2 IgGs was associated with a trend towards increased Caspr2 expression [145] as also observed in neuronal cell cultures in which incubation with anti-Caspr2 IgGs induce the clustering of Caspr2 at the cell surface and increase Kv1.2 expression [148]. Interestingly, in hippocampal cell culture, anti-Caspr2 antibodies from patients specifically target the GABAergic neurons. The inhibitory neurons, mainly of the parvalbumin and somatostatin subclasses, display high expression of the VGKC complex including Kv1.1/1.2, Caspr2, Contactin2, and ADAM22 at the AIS and along the axon at a pre-myelinating stage [57,59,149]. By increasing Kv1 expression, anti-Caspr2 autoantibodies could lead to reduced activity of inhibitory neurons, a defect consistent with seizure disorders observed in patients. Thus, defining the subcellular and cell-type-specific targeting of autoantibodies against LGI1 and Caspr2 may help to define their pathogenicity in the CNS.

The clinical characteristics of the patients indicate that only autoantibodies to Caspr2 are found associated with peripheral hyperexcitability including neuromyotonia and neuropathic pain [50,150,151,152]. The selective pathogenic mechanisms of autoantibodies against Caspr2 in peripheral nerves may be linked with its role in the clustering of Kv1 at the juxtaparanodes, at the soma of DRG neurons, or at the terminal endings of sensory and motor-axons. Most patients have dramatic responses to immunotherapy as an indication that the autoantibodies may likely display function-blocking activity rather than mediating neuronal destruction. Injection of anti-Caspr2 purified from patients causes pain-related hypersensitivity in mice. Caspr2 is co-localized with Kv1.1/1.2 channels at the soma of DRG neurons and the cell surface expression of Kv1 in DRG neurons is reduced following the passive transfer of anti-Caspr2 antibodies and in Caspr2-deficient mice. Such decreased Kv1 expression is associated with a large reduction of the dendrotoxin-sensitive outward K^+^ current resulting in enhanced intrinsic excitability [147]. The molecular mechanisms implicated in the modulation of Kv1 expression are not fully elucidated. Anti-Caspr2 autoantibodies that mainly belong to the IgG4 subclass do not cause Caspr2 internalization but prevent Caspr2/Contactin2 interaction as observed in a solid phase assay and in transfected HEK cells [148,153]. Anti-Caspr2 antibodies may be implicated in the cell surface mobility or clustering of the Kv1 complex, depending on Caspr2 interaction with Contactin2 and the submembrane scaffold. As another possible mechanism, anti-Caspr2 antibodies may trigger or inhibit downstream signaling pathways. For example, it was previously shown that mTOR signaling could suppress the translation of Kv1.1 and the mTOR inhibitor rapamycin increases Kv1.1 protein in the hippocampus [154]. Interestingly, sensory neurons in Caspr2 KO mice showed elevated Akt-mTOR signaling related to hyperexcitability and pain hypersensitivity [155].

## 9. Conclusions and Future Directions

In this review, we analyzed the trafficking and clustering of the Kv1 channels at the distinct axonal domains constituted by the AIS and juxtaparanodes. Specific molecular motors and adaptors are implicated in the anterograde axonal transport of Kv1 cargo and insertion at the cell membrane at specific sites. The mechanisms implicated in the clustering of Kv1 at the distal part of the AIS are still elusive. In myelinated axons, axo-glial cell adhesion molecules and the 4.1B cytoskeleton linker are implicated in the assembly of juxtaparanodes although the detailed molecular organization of the complex remains to be elucidated. The Kv1 channels are also restricted at the juxtaparanodes by the presence of paranodal junctions acting as a fence between the internodes and the nodes.

Recent findings indicate that myelination is not uniform in the CNS. Myelin organization and function may differ on long-range connecting axons in the white matter and the local circuitry of cortical grey matter. Strikingly, a large fraction of neocortical and hippocampal myelin ensheathes GABAergic axons in mice and humans [24,25]. Myelination of paravalbumin interneurons has been reported to shape the function of cortical sensory inhibitory circuits and to be specifically impaired in a rat model of schizophrenia [156,157]. Myelin displays a patchy distribution along the ramified axons of inhibitory parvalbumin basket cells [25] implying the presence of heminodes along the mature myelinated axons. Moreover, specific mechanisms have been reported for the clustering of Nav1 channels along GABAergic axons before myelination, which is induced by soluble oligodendroglial factors at the very beginning of myelination [158,159]. We recently observed that GABAergic neurons display a strong expression of Kv1 channels and Kv1-associated molecules at the AIS and along the axon at a premyelinated stage and that Kv1 clustering requires contact with the oligodendroglial membrane unlike nodal Nav1 channels [149]. Doubtless, evaluating the importance of myelin alteration of the GABAergic neurons in the pathophysiology of MS should deserve further investigation. We can hypothesize that the disruption of axo-glial contacts in demyelinated axons may lead to exposure of Kv1 channels altering spike propagation in the inhibitory axons. The K^+^ leak in the extracellular space may also have a deleterious effect through the activation of ectopic firing [160]. Since seizures may signal disease onset or relapse in a subset of MS patients [161,162] it could be further interesting to appreciate the involvement of excitatory/inhibitory balance at the early stages of the disease.
